# Theoretical Analysis of Superior Photodegradation of Methylene Blue by Cerium Oxide/Reduced Graphene Oxide vs. Graphene

**DOI:** 10.3390/molecules29163821

**Published:** 2024-08-12

**Authors:** Nguyen Hoang Hao, Phung Thi Lan, Nguyen Ngoc Ha, Le Minh Cam, Nguyen Thi Thu Ha

**Affiliations:** 1 College of Education, Vinh University, 182 Le Duan, Vinh 460000, Vietnam; haonguyen0404@gmail.com; 2 Faculty of Chemistry, Hanoi National University of Education, 136 Xuan Thuy, Cau Giay, Hanoi 100000, Vietnam; lanpt@hnue.edu.vn (P.T.L.); hann@hnue.edu.vn (N.N.H.); camlm@hnue.edu.vn (L.M.C.); 3 Faculty of Pharmacy, Thanh Do University, QL32, Kim Chung, Hoai Duc, Hanoi 100000, Vietnam

**Keywords:** CeO_2_, rGO, graphene, DFT+U, methylene blue, GFN-xTB

## Abstract

Density functional theory and a semi-empirical quantum chemical approach were used to evaluate the photocatalytic efficiency of ceria (CeO_2_) combined with reduced graphene oxide (rGO) and graphene (GP) for degrading methylene blue (MB). Two main aspects were examined: the adsorption ability of rGO and GP for MB, and the separation of photogenerated electrons and holes in CeO_2_/rGO and CeO_2_/GP. Our results, based on calculations of the adsorption energy, population analysis, bond strength index, and reduced density gradient, show favorable energetics for MB adsorption on both rGO and GP surfaces. The process is driven by weak, non-covalent interactions, with rGO showing better MB adsorption. A detailed analysis involving parameters like fractional occupation density, the centroid distance between molecular orbitals, and the Lewis acid index of the catalysts highlights the effective charge separation in CeO_2_/rGO compared to CeO_2_/GP. These findings are crucial for understanding photocatalytic degradation mechanisms of organic dyes and developing efficient photocatalysts.

## 1. Introduction

Dye pollutants stand out as persistent and problematic contaminants in water bodies within the context of environmental issues [1]. Photocatalysis, which uses catalysts and light to degrade dyes into harmless compounds, offers a promising solution for sustainable dye treatment and has emerged as an effective method for addressing this problem [2].

Among the variety of photocatalytic materials, ceria (cerium oxide, CeO_2_) is considered one of the most promising for dye treatment due to its high efficiency, non-toxicity, photochemical stability, affordability, and its excellent ability to switch between Ce^3+^ and Ce^4+^ states, thereby enhancing catalytic efficiency in various reactions. However, ceria catalysts also exhibit limitations that hinder their practical applications. One significant drawback is their typically small specific surface area, which can limit photocatalytic efficiency. More critically, ceria suffers from a high rate of electron–hole recombination, a major challenge in photocatalysis, as this recombination reduces the number of charge carriers available for the photocatalytic reaction. A potential solution to this problem involves combining ceria with materials that have large specific surface areas and can rapidly conduct electrons, thereby reducing the recombination of photogenerated electrons and holes (e* and h^+^). Examples of such materials include reduced graphene oxide (rGO) and graphene (GP) [3,4,5]. The large surface area of GP and rGO provides ample active sites for the adsorption of dye molecules, thereby enhancing the efficiency of the photocatalytic process through increased catalyst–dye contact [6,7]. Furthermore, the excellent electron transport properties of graphene-based materials facilitate efficient electron transfer, leading to the generation of reactive oxygen species crucial for organic compound degradation under light irradiation [3,8].

It is noteworthy that the number of research projects on CeO_2_/rGO significantly exceeds those on CeO_2_/GP. Besides the fact that rGO is easier to synthesize than GP, structural differences—particularly the presence of oxygen-containing functional groups and distinct electronic properties between rGO and GP—may also account for the differences in their ability to adsorb organic dyes and the optical properties of rGO-based photocatalysts compared to GP-based photocatalysts [9,10]. The photocatalytic degradation of organic dyes is a complicated process comprising numerous stages, with the initial step involving the adsorption of dye molecules onto the surface of the photocatalyst. The photocatalytic efficiency of CeO_2_/rGO and CeO_2_/GP can be directly impacted by the dye adsorption capacity. The surface of rGO has demonstrated a greater affinity for adsorbing cationic dyes compared to anionic dyes [11]. In a study conducted by Mayara et al., utilizing a combination of multi-scale simulations and experiments, the authors demonstrated that the adsorption of methylene blue (MB) onto graphene oxide flakes is primarily of a physical nature and dependent on the functional groups on the surface [12]. In addition to the surface area and the presence of oxygen-containing functional groups, the existence of π-π and electrostatic interactions further enhances the adsorption capacity of rGO for cationic dyes like MB [13,14,15]. The adsorption capacity of MB on rGO has been documented to reach values in the range of 2000 mg/g [16], whereas the adsorption capacity of MB on GP typically amounts to approximately 200 mg/g [17], indicating the superior adsorption capacity of rGO over GP for MB.

In this study, we utilized computational chemistry methods to delve into the molecular-level reasons behind the superior photocatalytic performance of CeO_2_/rGO over CeO_2_/GP. Our focus was on two key aspects: (i) understanding how MB sticks to rGO and GP, shedding light on the forces driving the adsorption process; and (ii) elucidating the differences in electronic structure between CeO_2_/rGO and CeO_2_/GP. These findings aim to provide a clear and thorough comparison of the photocatalytic abilities of CeO_2_/rGO and CeO_2_/GP.

## 2. Results and Discussions

### 2.1. Adsorption Behavior of Graphene and Reduced Graphene Oxide toward Methylene Blue

**Figure 1:**
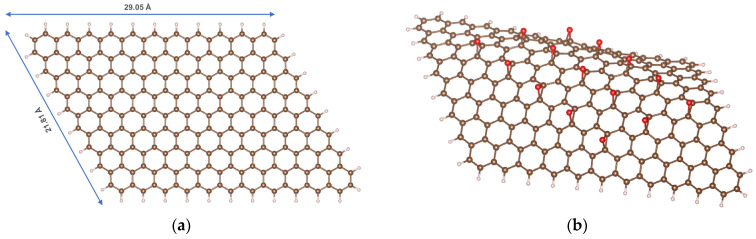
Models of GP (**a**) and rGO (**b**); color codes: brown—C; ivory—H; red—O.

A graphene model is constructed as a flat carbon layer containing 214 atoms, where the boundary carbon atoms are saturated with hydrogen atoms, corresponding to the formula C_214_H_40_ (Figure 1a). The mass fraction of carbon in this model is 98.5%, consistent with experimental analyses of the chemical composition of GP [18,19]. It should be noted that, in reality, oxygen always constitutes a small proportion of the chemical composition of GP. However, for ideal modeling purposes, we employ a completely oxygen-free GP model.

The reduced graphene oxide (rGO) model was derived from the GP model by adding the epoxide functional groups, resulting in the general formula C_214_H_40_O_15_ (Figure 1b). The mass fractions of carbon, hydrogen, and oxygen were 90.2%, 1.40%, and 8.43%, respectively, aligning with experimental studies [19]. Although the functional groups on the surface of rGO can be quite diverse, previous studies have demonstrated that epoxides are unique among oxygen-containing functional groups in their ability to tune the band gap. In contrast, other oxygen-containing groups are less effective: hydroxyls do not alter the band gap, and carbonyl and carboxyl groups disrupt the hexagonal carbon-ring structure of rGO [20,21].

The adsorption of MB on GP and rGO was evaluated by calculating adsorption energy values (E_ads_) and conducting a detailed population analysis. This analysis encompassed charge transfer (q), Wiberg bond orders (BOs), molecular orbital interactions, and various other non-covalent interactions.

The adsorption energy is calculated using the following formula:

E_ads_ = E_*adsorbent+MB*_ − E_*adsorbent*_ − E_*MB*_, (1)

where E_*adsorbent+MB*_ is the energy of the MB adsorption configuration on the GP or rGO adsorbent, E_*adsorbent*_ is the energy of the adsorbent, and E_*MB*_ is the energy of the MB molecule.

**Figure 2:**
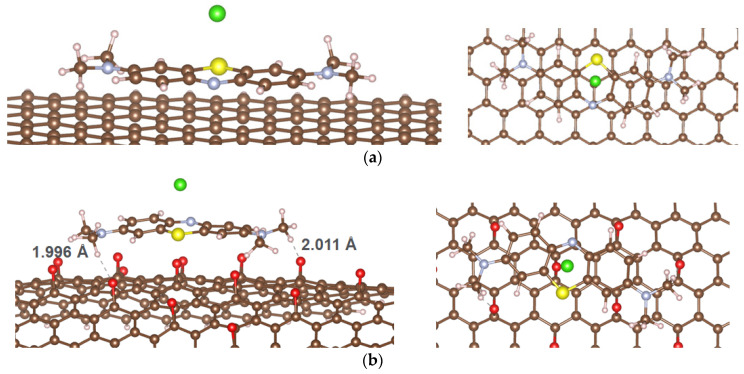
Optimized adsorption configurations of MB on GP (**a**) and rGO (**b**); color codes: brown—C; ivory—H, yellow—Ce; red—O; green—Cl; gray—N; light yellow—S.

The optimized adsorption configurations of MB on GP and rGO are shown in Figure 2, and the calculated results are presented in Table 1.

The results demonstrate that the adsorption of MB on both GP and rGO is energetically favorable, as indicated by negative adsorption energy values. However, rGO exhibits a superior adsorption ability due to its significantly lower E_ads_. The optimal adsorption configuration of MB is a parallel orientation on the adsorbent surface, consistent with previous studies [22]. This alignment is influenced by π-π stacking interactions, which are critical in the adsorption of aromatic organic compounds on carbon materials [23,24]. Despite substantial charge transfer from the MB molecule to the adsorbent, the calculations indicate that the minimal distance from MB to the adsorbent surface and the Wiberg bond order do not suggest the formation of chemical bonds between MB and the surfaces of GP or rGO. For context, the covalent radii of carbon (C), hydrogen (H), and oxygen (O) are 0.76, 0.31, and 0.66 Å [25], respectively, while their van der Waals (vdW) radii are 1.7, 1.20, and 1.52 Å [26], respectively. Thus, the minimal distance from MB to GP or rGO is significantly larger than the sum of the covalent radii of the atoms but smaller than the sum of the vdW radii. This raises questions about the nature of the interaction force between MB and the adsorbent surfaces, given the relatively negative adsorption energy values. It is important to note that the calculated Eads differs from the experimental heat of adsorption determined from adsorption isotherms, as the calculations only consider the interaction between an MB molecule and the adsorbent surface. Due to the large size of the MB molecule and its nature as a cationic dye, the vdW and other weak interactions, as well as electrostatic interactions, are likely to play a significant role. Although the Wiberg bond order between MB and both GP and rGO is negligible, this value only accounts for covalent bonding, not other types of interactions. Therefore, we proceeded to calculate the intrinsic bond strength index (IBSI) [27] to quantify the strength of chemical bonds. The IBSI can also be used to compare the strength of weak interactions. The IBSI is expressed as

(2) $$IBSI=\frac{(1/d^{2})\int \delta g^{pair}dr}{(1/d_{H_{2}}^{2})\int \delta g^{H_{2}}dr}$$

where *d* is the distance between the two atoms for which the interaction is being studied. The integral in the numerator is equivalent to the atomic pair δ*g* index defined between the two atoms. The denominator represents the reference system data, the $d_{H_{2}}$ and the integral are the bond length and atomic pair δ*g* index of *H*_2_ in its equilibrium structure, respectively.

**Figure 3:**
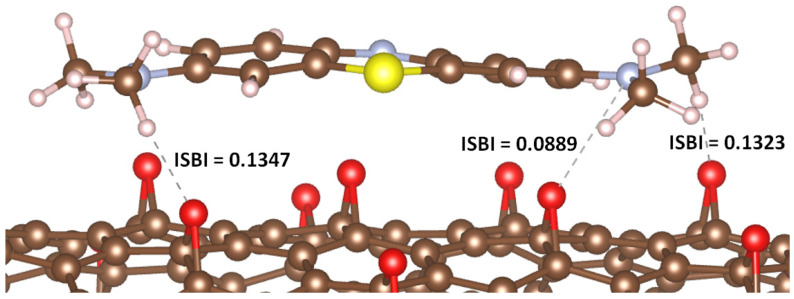
IBSI values corresponding to the interatomic interactions between atoms of MB and atoms of rGO.

The results obtained are intriguing. In the case of MB adsorption on GP, no significant IBSI value was observed between the MB atoms and the carbon atoms on the GP surface. Conversely, when adsorbed onto rGO, the hydrogen and nitrogen atoms of MB interacted with the oxygen atoms in the epoxide groups of rGO, yielding relatively high IBSI values (see Figure 3).

**Figure 4:**
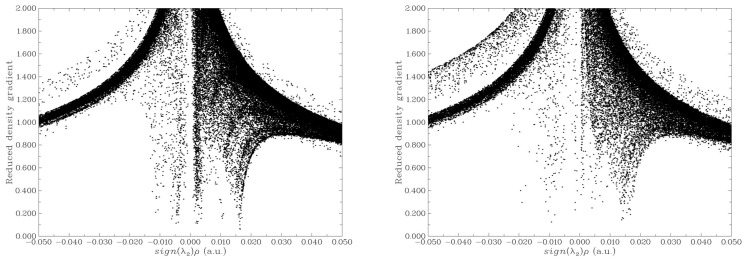
Scatter graph of RDG for MB/GP (**a**) and MB/rGO (**b**) systems.

**Figure 5:**
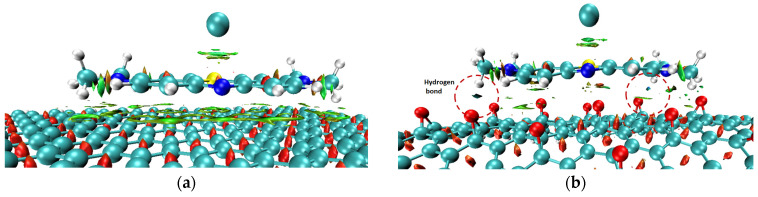
RDG isosurfaces (isovalue = 0.8) of MB/GP (**a**) and MB/rGO (**b**) systems.

Reduced density gradient (RDG) analysis was conducted to elucidate the weak intermolecular interactions between MB and GP, as well as between MB and rGO. The resulting map, which shows the dependence of RDG on sign (λ_2_) ρ (where λ_2_ is the second largest eigenvalue of the Hessian matrix of electron density and ρ is the electron density), is presented in Figure 4 for both the MB/GP and MB/rGO systems. Figure 5 depicts the RDG isosurfaces of the adsorption configurations.

In the case of MB/GP, the RDG graph (Figure 4a) shows spikes in the range of sign (λ_2_) ρ values from −0.01 to 0.015 a.u, corresponding to vdW interactions between the MB molecule and the GP surface (colored in green in Figure 5a). Due to the flat structure of the GP surface, the vdW interactions and other weak interactions, such as π-π stacking, between MB and GP are more pronounced than on the curved surface of rGO. However, when interacting with rGO, in addition to vdW interactions similar to those with GP, there are also regions indicating hydrogen bond formation (colored in blue in Figure 5b). Additionally, the interaction between the N-H groups of MB and the oxygen atoms of rGO through hydrogen bonds or electrostatic interactions aligns well with the findings of Manash et al. [15]. Consequently, calculations of adsorption energy, along with structural parameter and population analysis, demonstrate that the adsorption of MB on rGO is more favorable than on GP.

### 2.2. Interaction between Graphene and Reduced Graphene Oxide with Ceria Cluster

The CeO_2_-GP and CeO_2_-rGO models were constructed by placing (CeO_2_)_6_ clusters on the surfaces of GP and rGO. The CeO_2_ cluster model was selected to simulate small-sized CeO_2_ nanoparticles dispersed on the surfaces of GP and rGO. Similar approaches using CeO_2_ clusters have been employed in studies of other surface catalytic reactions [28,29].

As previously indicated, rGO demonstrates a significantly superior MB adsorption ability compared to GP due to the presence of oxygen-containing functional groups. Consequently, utilizing rGO enhances the concentration of dyes on the surface of the catalytic system, thereby creating favorable conditions for subsequent conversions at the photocatalytic centers, including ceria or rGO/MB itself. Further, we estimate the interaction of GP and rGO with ceria clusters. Effective interaction between the components in the CeO_2_/GP or CeO_2_/rGO catalytic system is anticipated to enhance photocatalytic activity by promoting the efficient release of photogenerated electrons and holes.

First, we calculated the interaction energy (E_int_) between the (CeO_2_)_6_ cluster and both GP and rGO. Eint is determined using the following formula:

E_int_ = E_*adsorbent+cluster*_ − E_*adsorbent*_ − E_*cluster*_, (3)

where E*_adsorbent+cluster_* is the energy of (CeO_2_)_6_/GP or (CeO_2_)_6_/rGO, and E*_cluster_* is the energy of the ceria cluster.

**Figure 6:**
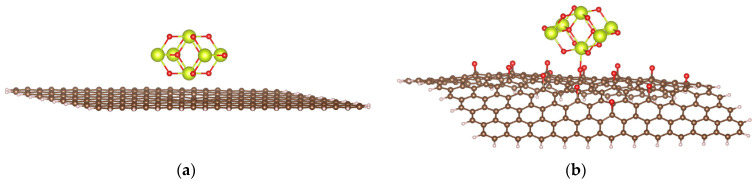
Optimized structures of CeO_2_/GP (**a**) and CeO_2_/rGO (**b**).

The optimized structures of CeO_2_/GP and CeO_2_/rGO are shown in Figure 6. The .xyz files of these optimized structures are presented in the Supplementary Materials. Calculation results show that the interaction between (CeO_2_)_6_ and GP has an E_int_ value of +108.48 kJ mol^–1^, while the interaction between this cluster and rGO releases 32.63 kJ mol^–1^ of energy. These findings indicate that the interaction between the ceria cluster and the GP surface is much less energetically favorable than the interaction between the cluster and rGO. The presence of oxygen-containing functional groups on the surface of rGO allows it to interact more favorably with metal-based catalysts. This result aligns well with previous experimental studies. In the work of Pandey et al. on novel three-phase polymer nanocomposites based on cerium oxide (CeO_2_) nanoparticles and graphene nanoplatelets (GNPs) incorporated in poly (vinylidene fluoride) (PVDF) [30], the authors pointed out that the addition of GNPs in the PVDF/CeO_2_ nanocomposites did not cause any notable change in the FTIR spectra, indicating weak or no interaction between CeO_2_ and GP.

On the other hand, the interaction between CeO_2_ and rGO is energetically favorable, as evidenced by the negative E_int_ value. In fact, CeO_2_/rGO material systems are widely studied in photocatalytic applications [31,32].

**Figure 7:**
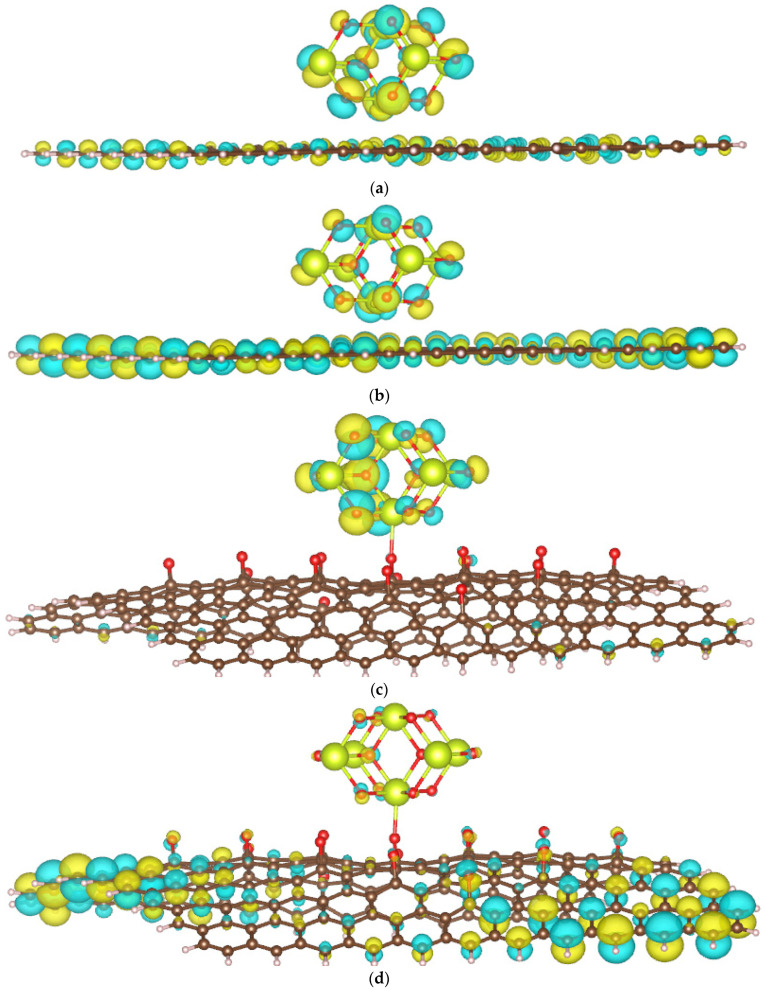
Frontier molecular orbitals of CeO_2_/GP: HOMO (**a**) and LUMO (**b**); and CeO_2_/rGO: HOMO (**c**) and LUMO (**d**).

Due to the complexity of calculating the bandgap values for the studied materials, particularly because of the presence of an f-element (Ce), we instead evaluate the photocatalytic ability by examining the spatial distribution of HOMOs and LUMOs, as discussed in [33]. A greater spatial separation between HOMOs and LUMOs may correlate with a lower recombination rate of photogenerated electrons and holes. Figure 7 presents the frontier molecular orbitals, including the HOMO and LUMO, for the CeO_2_/GP (a) and CeO_2_/rGO (b) systems.

In the CeO_2_/GP system, both the ceria cluster and the carbon atoms of GP mainly contribute to the HOMO and LUMO. In contrast, in the CeO_2_/rGO system, the HOMO is primarily located on the ceria cluster, while the LUMO is mainly contributed by rGO. This distribution suggests that the HOMO-LUMO configuration of CeO_2_/rGO will be more favorable for reducing the recombination of photogenerated electrons and holes compared to CeO_2_/GP.

**Figure 8:**
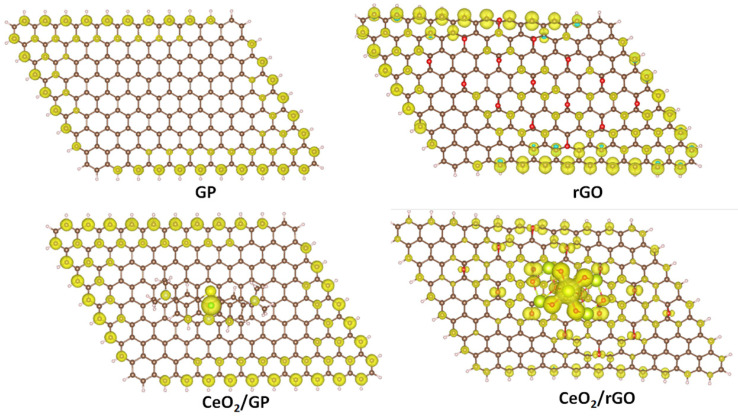
The FOD maps of GP, rGO, CeO_2_/GP, and CeO_2_/rGO.

We further calculated the fractional occupation number weighted density (FOD) to quickly and robustly provide information on the localization of “hot” (strongly correlated and chemically active) electrons in catalytic systems. The FOD map of GP and rGO (Figure 8) reveals that in the absence of CeO_2_, the active electrons are concentrated on the carbon atoms on the surface of the material. In the CeO_2_/GP and CeO_2_/rGO systems, active electrons are also distributed on the ceria cluster, which is particularly noticeable in the case of CeO_2_/rGO. This finding is interesting when correlated with the HOMO and LUMO images of CeO_2_/rGO (Figure 7c,d). The active electrons are primarily located in the HOMO of the ceria cluster, which can be excited and transferred to the LUMO located on rGO, thereby reducing the recombination ability.

To facilitate a more detailed analysis of charge transfer during electron excitation, we calculated the centroid distance between the HOMO and the LUMO of the photocatalytic systems. The centroid distance (*D_ij_*) was determined as follows:

(4) $$D_{ij}=\sqrt{{\left(X_{i}-X_{j}\right)}^{2}+{\left(Y_{i}-Y_{j}\right)}^{2}+{\left(Z_{i}-Z_{j}\right)}^{2}}$$

where *X*, *Y*, and *Z* are the coordinates of the centroid of the orbitals. The calculated results are presented in Table 2, along with parameters related to the electronic properties including absolute electronegativity (χ), the global electrophilicity index (GEI) of the photocatalytic systems, and the band gap of CeO_2_/GP and CeO_2_/rGO. Due to the complexities involved in calculating the UV-VIS spectra for large systems containing f-elements such as Ce, we have employed an alternative approach based on IP and EA values to evaluate the transport band gap of the studied system. According to this method, the transport band gap (E_g_) is defined as E_g_ = IP − EA [34,35].

The computational results indicate that the calculated E_g_ values of CeO_2_/GP and CeO_2_/rGO are relatively close to each other and smaller than the experimental values. It is important to note that, due to the size limitations of the model, the calculated band gap values for the studied systems are intended to assess the trends in variation rather than provide absolute values. Experimental measurements reveal that the optical band gap values of CeO_2_/GP and CeO_2_/rGO are also relatively similar, approximately 2.7 eV [31,36]. Therefore, from the standpoint of light absorption capability, there is no significant difference between CeO_2_/GP and CeO_2_/rGO. Consequently, the variation in photocatalytic activity is likely primarily attributed to the recombination rates of photogenerated electrons and holes.

The *Dij* value of the material systems increases in the following order: (CeO_2_)_6_ < GP < CeO_2_/GP < rGO < CeO_2_/rGO. This indicates that combining ceria with GP and rGO can significantly reduce the recombination ability of photogenerated electrons and holes. Among the studied material systems, the CeO_2_/rGO system exhibits the best ability to separate e*–h^+^.

The incorporation of ceria into GP and rGO increases both the absolute electronegativity value and the global electrophilicity index (GEI) compared to the original materials. Notably, the GEI can be used as a measure of Lewis acidity [37,38]. Previous studies have highlighted a positive correlation between Lewis acidity, or the number of Lewis acid centers, and the photocatalytic decomposition activity of methylene blue and rhodamine B in photocatalysts [39,40]. Therefore, the increased GEI value of material systems due to the presence of ceria is favorable for the decomposition of methylene blue.

## 3. Computational Methods

All geometry calculations were performed using density functional theory (DFT) methods, as implemented in the CP2K code (https://www.cp2k.org/). The exchange-correlation potential was described using the generalized gradient approximation (GGA) with the Perdew–Burke–Ernzerhof (PBE) spin-polarized functional [41]. Wavefunctions were expanded using a double-zeta valence polarized (DZVP) basis set, supplemented by an auxiliary plane-wave basis with a cutoff energy of 250 Ry. Core electrons were modeled using norm-conserving pseudopotentials [42]. Brillouin zone integration was performed using a reciprocal space mesh that included only the Γ-point. Dudarev’s approach for DFT+U calculations was employed to describe the Ce 4f electrons [43]. It should be noted that the bandgap value of the bulk CeO_2_ varies from 2.94 to 3.42 eV [44]. However, this value varies significantly for the (CeO_2_)_6_ cluster model due to the influence of cluster structures and sizes. In this study, a U–J value of 16.0 eV was used, corresponding to the highest bandgap value simulated for the (CeO_2_)_6_ cluster, which is 1.99 eV. Additionally, van der Waals forces were included using DFT-D calculations based on the Grimme D3 method to accurately estimate interaction forces [45].

The electronic properties of the studied systems, specifically the vertical ionization energy (IP), vertical electron affinity (EA), and global electrophilicity index (GEI), were determined through single-point calculations utilizing the GFN1-xTB method [46,47]. Non-covalent interactions were analyzed using Multiwfn software v3.8 [48].

## 4. Conclusions

The superior photocatalytic efficacy of CeO_2_/rGO over CeO_2_/GP in the photodegradation of methylene blue has been demonstrated through the evaluation of MB adsorption on the rGO substrate relative to GP, alongside an examination of the electronic characteristics inherent to these catalytic systems. The calculated adsorption energy and population analysis reveal that the adsorption of MB onto both GP and rGO is energetically favorable, primarily driven by non-covalent interactions, including van der Waals forces, π-π stacking interactions, and electrostatic interactions. Notably, rGO exhibits a heightened MB adsorption capacity relative to GP, attributable to the formation of weak bonds such as hydrogen bonds with oxygen atoms on its surface. The calculated interaction energies indicate that the combination of ceria with GP lacks energetic favorability compared to rGO. Through a comprehensive assessment of frontier molecular orbitals, fractional occupation density, the centroid distance between molecular orbitals, and the Lewis acid index of the catalysts, it has been demonstrated that CeO_2_/rGO exhibits superior efficacy in the separation of photogenerated electrons and holes compared to CeO_2_/GP. These findings hold substantial implications for understanding the mechanisms governing the photocatalytic degradation of organic dyes, as well as for the development of novel effective photocatalysts.

## Figures and Tables

**Table 1:** Calculated results for the adsorption of MB on GP and rGO.

Parameter	E_ads_, kJ mol^−1^	d_min_, Å	q(MB), au	BO
MB/GP	−102.72	2.640 (H–C)	0.249	<0.10
MB/rGO	−405.78	1.996 (H···O)	0.411	<0.10

Note for Table 1: d_min_ is the minimal distance from the adsorbed MB molecule to the surface of the adsorbent.

**Table 2:** Electronic properties and E_g_ of (CeO_2_)_6_, GP, rGO CeO_2_/GP, and CeO_2_/rGO.

System	D_ij_, Å	χ, eV	GEI, eV	E_g_, eV
(CeO_2_)_6_	0.023	5.3792	3.4417	-
GP	0.029	5.0983	8.7913	-
rGO	8.181	5.4622	10.0683	-
CeO_2_/GP	3.472	8.7657	26.6079	1.4439
CeO_2_/rGO	9.666	7.6218	20.7440	1.4001

## Data Availability

The data that support the findings of this study are available from the corresponding author upon reasonable request.

## Supplementary Materials

The following supporting information can be downloaded at: https://www.mdpi.com/article/10.3390/molecules29163821/s1. Coordinates of atoms for the optimized CeO_2_/rGO and CeO_2_/GP structures.
