# Supplementary material for: Theoretical Analysis of Superior Photodegradation of Methylene Blue by Cerium Oxide/Reduced Graphene Oxide vs. Graphene
Source: Molecules. 2024 Aug 12;29(16):3821. doi: 10.3390/molecules29163821 (PMC11356922; doi:10.3390/molecules29163821)
Supplement: Supplementary file 1 [file molecules-29-03821-s001.zip › molecules-3130686-supplementary.pdf]

SUPPLEMENTARY INFORMATION

Coordinates of atoms for the optimized CeO_2/rGO and CeO_2/GP structures

<u>CeO_2/rGO</u>

6	28.639287800	14.168415330	11.642620670
6	32.412221800	14.824304330	11.677044670
6	24.904646800	14.851450330	11.254708670
6	36.164394800	14.201510330	11.534610670
6	24.913601800	16.391024330	11.146870670
6	29.930288800	14.880360330	11.505855670
6	31.127566800	14.115076330	11.520287670
6	27.383866800	14.891229330	11.333439670
6	33.690233800	14.114588330	11.476773670
6	28.638287800	12.621335330	11.456500670
6	21.153901800	14.236384330	11.378231670
6	21.187373800	17.115181330	11.370681670
6	26.191146800	14.137152330	11.192416670
6	17.413498800	16.483746330	11.407061670
6	32.408057800	16.370853330	11.377285670
6	36.163870800	12.614580330	11.350727670
6	28.661545800	17.036348330	11.327389670
6	22.431658800	14.944795330	11.211882670
6	34.879629800	14.879990330	11.379237670
6	27.388907800	16.303228330	11.216584670
6	32.408115800	11.973547330	11.327642670
6	23.615136800	14.170927330	11.106945670
6	22.439094800	16.358427330	11.147224670
6	31.125801800	12.707247330	11.337524670
6	19.894821800	16.402613330	11.287564670
6	29.936378800	16.287195330	11.308097670
6	33.691414800	12.706365330	11.304103670

6	17.413978800	14.923373330	11.253819670
6	19.885318800	14.982897330	11.236233670
6	18.705088800	17.170784330	11.216039670
6	26.209821800	17.051725330	10.972590670
6	23.634726800	17.089010330	10.943527670
6	37.423571800	14.896495330	11.203200670
6	26.177349800	12.736839330	11.098199670
6	38.617819800	14.153980330	11.170405670
6	13.672684800	17.115665330	11.112541670
6	16.144534800	17.183907330	11.121325670
6	24.888388800	12.032188330	11.036932670
6	29.928083800	11.971049330	11.133795670
6	34.879967800	11.984448330	11.047168670
6	21.201734800	18.637068330	11.047777670
6	23.618512800	12.776014330	10.950388670
6	14.957511800	16.420629330	11.041616670
6	27.375224800	11.982631330	11.023212670
6	31.126197800	17.007272330	11.053357670
6	18.698345800	14.247169330	11.032486670
6	21.150568800	12.721571330	11.022071670
6	34.871159800	16.260564330	11.019758670
6	38.639787800	12.741260330	10.990120670
6	18.720039800	18.561323330	10.925260670
6	13.684891800	18.688799330	10.872051670
6	37.406584800	12.012855330	10.878990670
6	17.447580800	19.306026330	10.875339670
6	16.156240800	18.587158330	10.881857670
6	33.651957800	16.965349330	10.871891670
6	23.656552800	18.475245330	10.718206670
6	37.353567800	16.247002330	10.837325670

6	14.961088800	15.006171330	10.814620670
6	28.672856800	18.499533330	10.784138670
6	16.179670800	14.282410330	10.782856670
6	12.431444800	16.410578330	10.775767670
6	26.212434800	18.419705330	10.645841670
6	32.403704800	10.541369330	10.698510670
6	24.945484800	19.167343330	10.617080670
6	39.847220800	12.064744330	10.705178670
6	22.426771800	12.073645330	10.693136670
6	36.117978800	16.926815330	10.683582670
6	19.921809800	19.268404330	10.669691670
6	11.225261800	17.134770330	10.622902670
6	14.978724800	19.304420330	10.579922670
6	22.467251800	19.223685330	10.561150670
6	29.934816800	10.658516330	10.585712670
6	18.702961800	12.886526330	10.582712670
6	12.475381800	15.012652330	10.563331670
6	13.727208800	14.316920330	10.530932670
6	27.374813800	10.672725330	10.489993670
6	9.981852800	16.406983330	10.479037670
6	24.900344800	10.598796330	10.440368670
6	34.880953800	10.691607330	10.424696670
6	19.917111800	12.161806330	10.461158670
6	11.231256800	18.560977330	10.413739670
6	31.128947800	18.302936330	10.438557670
6	28.653030800	9.941653330	10.384215670
6	10.020235800	14.998645330	10.387961670
6	27.406626800	19.094392330	10.341515670
6	12.452644800	19.282760330	10.351934670
6	8.771075800	17.122714330	10.341348670

6	11.240507800	14.292278330	10.335496670
6	37.375309800	10.733114330	10.283879670
6	16.225123800	12.948611330	10.308574670
6	31.126960800	10.007253330	10.207196670
6	39.858627800	10.744758330	10.198087670
6	33.611183800	18.226919330	10.231067670
6	17.471856800	20.709127330	10.166323670
6	29.918133800	18.996675330	10.193576670
6	33.659824800	10.034834330	10.128899670
6	17.475928800	12.259177330	10.174034670
6	8.755864800	18.519995330	10.132625670
6	13.758282800	12.950927330	10.134969670
6	36.068228800	18.209110330	10.106258670
6	10.013983800	19.227515330	10.077385670
6	36.119044800	10.082909330	10.026484670
6	26.194083800	10.047708330	10.028423670
6	22.441822800	10.800371330	10.051434670
6	11.295683800	12.918239330	10.016153670
6	19.930222800	20.539322330	10.020993670
6	15.003966800	12.280839330	9.962768670
6	32.365604800	18.882702330	9.980881670
6	38.601250800	10.092615330	9.925546670
6	14.994097800	20.582935330	9.921783670
6	41.061680800	10.065974330	9.894125670
6	7.558797800	19.230863330	9.892510670
6	24.948076800	20.536235330	9.858858670
6	22.480605800	20.478079330	9.900051670
6	12.523810800	12.246410330	9.850654670
6	34.846895800	18.849852330	9.812359670
6	23.666900800	10.129759330	9.799736670

6	12.504553800	20.576985330	9.773506670
6	19.967548800	10.911936330	9.791053670
6	21.215045800	21.204032330	9.726591670
6	10.039683800	20.582663330	9.631250670
6	18.746226800	21.177616330	9.614803670
6	16.218680800	21.203878330	9.591975670
6	17.506145800	10.962752330	9.582790670
6	7.561866800	20.592515330	9.511536670
6	13.765135800	21.207997330	9.526354670
6	28.663944800	8.682803330	9.499633670
6	27.404740800	20.282069330	9.545391670
6	21.216065800	10.240913330	9.562822670
6	11.288416800	21.235739330	9.421061670
6	15.038229800	10.954109330	9.439161670
6	23.663088800	21.036913330	9.372716670
6	31.144179800	8.839288330	9.366292670
6	12.581494800	10.919763330	9.368644670
6	8.819902800	21.264473330	9.334380670
6	33.636636800	8.858393330	9.333751670
6	41.065113800	8.783913330	9.301091670
6	36.108106800	8.841859330	9.321071670
6	29.881011800	20.152772330	9.364643670
6	18.751643800	10.310144330	9.340058670
6	38.584023800	8.813412330	9.297038670
6	6.360468800	21.311556330	9.254047670
6	16.284029800	10.313498330	9.202867670
6	32.344454800	20.088566330	9.218387670
6	26.190062800	20.911514330	9.184536670
6	26.203807800	8.896056330	9.174351670
6	13.806421800	10.271200330	9.119057670

6	34.797521800	20.073030330	9.100812670
6	42.272594800	8.093081330	8.996125670
6	39.811353800	8.158063330	8.976894670
6	28.641428800	20.778726330	9.019936670
6	37.338133800	8.204280330	8.965578670
6	34.867865800	8.250464330	8.942475670
6	23.718021800	8.985660330	8.965210670
6	32.390864800	8.272303330	8.941353670
6	29.939468800	8.246749330	8.925482670
6	8.854109800	22.615989330	8.843733670
6	11.323056800	22.559912330	8.847216670
6	6.401359800	22.638950330	8.816144670
6	31.107666800	20.695750330	8.867106670
6	27.424114800	8.278571330	8.824244670
6	13.792963800	22.465526330	8.820125670
6	21.250960800	9.034810330	8.801632670
6	33.582725800	20.678980330	8.764303670
6	16.252770800	22.352276330	8.733141670
6	18.785473800	9.051261330	8.660424670
6	7.621329800	23.286262330	8.601157670
6	24.973782800	8.377517330	8.644363670
6	18.733464800	22.246738330	8.634873670
6	10.103492800	23.227530330	8.593384670
6	21.211431800	22.352875330	8.621211670
6	16.319318800	9.027343330	8.580091670
6	12.576420800	23.128325330	8.531219670
6	13.866729800	8.978086330	8.539077670
6	22.498676800	8.428800330	8.472174670
6	42.243326800	6.835715330	8.385772670
6	15.035543800	22.985792330	8.397591670

6	20.030466800	8.435307330	8.359140670
6	23.661294800	22.048154330	8.339073670
6	39.792062800	6.875636330	8.326347670
6	17.563731800	8.412961330	8.281100670
6	37.324061800	6.945249330	8.260380670
6	17.494840800	22.824099330	8.252834670
6	15.086709800	8.359783330	8.242010670
6	26.151403800	21.937153330	8.185411670
6	34.860192800	7.028438330	8.172879670
6	32.395318800	7.108741330	8.089681670
6	28.619841800	21.884046330	8.098090670
6	41.029839800	6.230245330	8.046702670
6	19.927082800	22.684655330	8.045700670
6	31.082908800	21.860701330	8.018987670
6	29.936963800	7.153431330	7.995991670
6	38.550299800	6.304133330	7.971669670
6	33.529129800	21.866299330	7.958971670
6	27.462633800	7.194260330	7.886519670
6	36.084002800	6.394915330	7.867139670
6	22.441124800	22.570133330	7.886455670
6	24.901136800	22.462167330	7.774119670
6	25.000106800	7.235992330	7.765500670
6	33.624788800	6.503376330	7.742831670
6	22.530265800	7.233665330	7.664023670
6	27.382266800	22.409969330	7.667596670
6	29.855311800	22.407709330	7.630054670
6	20.064148800	7.198594330	7.617101670
6	31.172559800	6.593206330	7.610685670
6	17.601731800	7.143120330	7.601187670
6	15.154112800	7.076477330	7.598742670

6	32.331706800	22.424865330	7.587856670
6	28.709117800	6.633922330	7.517368670
6	26.243768800	6.686157330	7.384891670
6	23.779072800	6.684071330	7.307802670
6	16.358173800	6.498394330	7.282057670
6	21.309538800	6.639645330	7.267807670
6	18.837396800	6.576290330	7.267817670
1	39.569456800	14.698625330	11.077025670
1	40.800001800	12.601497330	10.821218670
1	38.280986800	16.773322330	10.566228670
1	7.824610800	16.564378330	10.303378670
1	9.078293800	14.445679330	10.257916670
1	42.021440800	10.556599330	10.113911670
1	6.598785800	18.699649330	9.968302670
1	37.010120800	18.700637330	9.821301670
1	10.358196800	12.374770330	9.827139670
1	5.396739800	20.802617330	9.399084670
1	43.231718800	8.566540330	9.249639670
1	11.641803800	10.395500330	9.140962670
1	35.739586800	20.537463330	8.773907670
1	5.461507800	23.177422330	8.627489670
1	12.927668800	8.460615330	8.293077670
1	7.644938800	24.324001330	8.238234670
1	10.128663800	24.247221330	8.180500670
1	43.187357800	6.317835330	8.164616670
1	12.611539800	24.107222330	8.031016670
1	15.058025800	23.904665330	7.794172670
1	34.477765800	22.312784330	7.628410670
1	41.015035800	5.245503330	7.557743670
1	17.505277800	23.675104330	7.557350670

1	38.534314800	5.334800330	7.451330670
1	14.211190800	6.567624330	7.352428670
1	36.069965800	5.450074330	7.304505670
1	33.614166800	5.596458330	7.121064670
1	19.897970800	23.332345330	7.158636670
1	24.885993800	23.236381330	6.994048670
1	32.304490800	23.322902330	6.954331670
1	29.841714800	23.270360330	6.948033670
1	22.402987800	23.174029330	6.969189670
1	27.373937800	23.220891330	6.925115670
1	31.183380800	5.727270330	6.933332670
1	28.725549800	5.778646330	6.827322670
1	16.393734800	5.522168330	6.777616670
1	18.862772800	5.618306330	6.728000670
1	21.335571800	5.709181330	6.682303670
1	26.263455800	5.830597330	6.694475670
1	23.807613800	5.791636330	6.665961670
8	24.870083800	15.702463330	12.397576670
8	28.570332800	13.251937330	12.763003670
8	32.465402800	15.820525330	12.712083670
8	36.247152800	13.278530330	12.622102670
8	17.331649800	15.593349330	12.535335670
8	21.259913800	18.125021330	12.406923670
8	21.097709800	13.212661330	12.393101670
8	28.687200800	18.177417330	12.205366670
8	13.561968800	18.081526330	12.167172670
8	32.439608800	10.779077330	12.129064670
8	24.798310800	10.858875330	11.861884670
8	17.450560800	20.543174330	11.606581670
8	25.025520800	20.425449330	11.299873670

8	28.611990800	8.618277330	10.961571670
8	21.286874800	22.589534330	10.066108670
8	23.632536800	16.281852330	16.609857670
8	24.154952800	18.057067330	14.636175670
8	23.110139800	14.506701330	18.583466670
8	21.015656800	16.964071330	16.530811670
58	22.808279800	18.472773330	16.507892670
58	21.840045800	14.773261330	16.632774670
8	25.803617800	13.675401330	14.840031670
8	24.078354800	18.206148330	18.458657670
8	23.186737800	14.357621330	14.760984670
58	24.979341800	15.866257330	14.738139670
8	26.771831800	17.374847330	14.715222670
8	25.727019800	13.824482330	18.662513670
8	28.866295800	14.917412330	16.767950670
8	26.249415800	15.599632330	16.688904670
58	24.902743800	16.015338330	18.560621670
58	28.042038800	17.108334330	16.665985670
58	27.073804800	13.408822330	16.790867670
8	26.695233800	17.523928330	18.537704670

b) CeO_2/GP

6	15.902549760	6.419210590	7.718457330
6	18.378239760	6.422452590	7.744584330
6	20.847913760	6.420023590	7.770210330
6	23.315202760	6.417305590	7.790701330
6	25.780053760	6.415997590	7.803061330
6	28.244171760	6.415360590	7.809359330
6	30.708794760	6.416738590	7.818309330
6	33.173147760	6.420133590	7.833193330
6	35.637009760	6.423723590	7.850210330

6	38.102435760	6.427354590	7.869079330
6	40.577686760	6.427547590	7.890948330
6	14.721156760	7.106090590	7.715005330
6	13.496255760	9.261387590	7.731278330
6	12.268438760	11.409742590	7.751336330
6	11.041917760	13.556136590	7.779195330
6	9.817409760	15.700621590	7.814760330
6	8.593054760	17.843135590	7.851160330
6	7.367060760	19.985798590	7.884907330
6	6.134579760	22.135743590	7.917865330
6	14.689188760	8.548634590	7.732131330
6	13.468529760	10.688117590	7.748120330
6	12.245055760	12.828777590	7.771571330
6	11.021832760	14.970717590	7.803993330
6	9.798425760	17.113104590	7.839961330
6	8.574185760	19.255370590	7.873688330
6	7.349851760	21.397249590	7.906429330
6	6.147023760	23.532944590	7.938062330
6	17.169458760	7.107154590	7.739985330
6	15.946025760	9.262476590	7.750911330
6	14.721175760	11.406811590	7.764349330
6	13.497051760	13.549036590	7.787078330
6	12.274200760	15.691026590	7.819273330
6	11.050854760	17.832903590	7.853885330
6	9.825340760	19.974375590	7.885718330
6	8.594543760	22.114660590	7.917034330
6	7.352456760	24.239091590	7.947685330
6	19.628547760	7.107038590	7.767139330
6	18.406970760	9.264066590	7.781992330
6	17.184505760	11.408828590	7.793369330

6	15.961467760	13.551070590	7.807929330
6	14.738805760	15.692530590	7.829102330
6	13.515998760	17.833283590	7.858061330
6	12.290977760	19.973612590	7.889877330
6	11.061809760	22.113237590	7.920019330
6	9.828363760	24.240128590	7.947962330
6	22.089484760	7.104228590	7.793834330
6	20.867990760	9.262587590	7.823541330
6	19.646884760	11.409045590	7.844758330
6	18.424568760	13.551670590	7.856777330
6	17.202038760	15.693575590	7.868176330
6	15.979748760	17.835161590	7.877424330
6	14.754780760	19.976168590	7.897539330
6	13.526678760	22.116730590	7.926790330
6	12.294484760	24.245102590	7.952787330
6	24.551200760	7.102574590	7.810834330
6	23.328480760	9.261504590	7.852151330
6	22.107407760	11.409738590	7.884641330
6	20.886834760	13.552392590	7.891590330
6	19.665499760	15.692802590	7.906761330
6	18.442059760	17.835869590	7.926099330
6	17.218001760	19.978539590	7.925204330
6	15.990788760	22.120758590	7.937351330
6	14.758934760	24.249838590	7.960963330
6	27.013696760	7.101140590	7.815895330
6	25.789380760	9.259958590	7.856926330
6	24.566483760	11.408902590	7.891348330
6	23.345423760	13.550513590	7.853660330
6	22.123352760	15.694339590	7.831749330
6	20.903963760	17.834051590	7.937756330

6	19.680710760	19.977970590	7.971228330
6	18.454744760	22.123177590	7.961239330
6	17.223910760	24.253777590	7.970295330
6	29.477063760	7.100982590	7.817592330
6	28.250635760	9.258775590	7.841780330
6	27.027236760	11.406131590	7.875860330
6	25.805316760	13.550334590	7.845861330
6	24.586413760	15.692913590	7.709365330
6	23.365090760	17.833840590	7.854494330
6	22.144452760	19.976454590	7.971553330
6	20.918047760	22.123449590	7.986817330
6	19.689358760	24.255974590	7.981522330
6	31.941029760	7.103400590	7.826734330
6	30.712566760	9.259412590	7.828846330
6	29.488508760	11.405778590	7.847034330
6	28.265643760	13.549463590	7.857953330
6	27.045419760	15.692247590	7.821588330
6	25.823600760	17.835125590	7.824914330
6	24.601784760	19.975806590	7.925314330
6	23.380711760	22.122618590	7.976884330
6	22.154451760	24.256257590	7.983539330
6	34.405233760	7.107464590	7.841486330
6	33.175251760	9.261965590	7.833195330
6	31.950188760	11.406894590	7.829522330
6	30.727702760	13.549499590	7.841657330
6	29.505992760	15.691102590	7.858077330
6	28.282109760	17.832554590	7.864672330
6	27.060678760	19.974787590	7.899755330
6	25.841983760	22.121155590	7.939289330
6	24.619665760	24.254784590	7.963121330

6	36.869336760	7.111555590	7.857382330
6	35.638977760	9.265460590	7.844801330
6	34.413369760	11.408919590	7.833489330
6	33.190958760	13.550908590	7.827427330
6	31.969418760	15.692210590	7.836179330
6	30.746832760	17.833110590	7.852711330
6	29.523813760	19.975179590	7.870415330
6	28.304267760	22.119164590	7.898115330
6	27.086864760	24.252113590	7.928630330
6	39.333855760	7.115820590	7.876224330
6	38.104793760	9.267482590	7.857644330
6	36.880084760	11.410503590	7.843386330
6	35.657765760	13.552547590	7.831322330
6	34.436231760	15.694402590	7.824422330
6	33.213504760	17.835642590	7.828473330
6	31.990182760	19.976317590	7.841863330
6	30.770116760	22.118093590	7.863185330
6	29.556884760	24.249445590	7.891928330
6	41.783198760	7.133423590	7.898108330
6	40.581627760	9.269806590	7.874910330
6	39.358988760	11.412855590	7.854715330
6	38.136263760	13.556296590	7.839058330
6	36.914025760	15.699724590	7.827315330
6	35.690794760	17.842270590	7.821654330
6	34.466870760	19.983101590	7.825957330
6	33.246017760	22.122964590	7.840419330
6	32.032872760	24.252943590	7.862782330
6	17.165156760	8.553776590	7.756145330
6	15.945066760	10.694957590	7.766548330
6	14.722188760	12.835551590	7.783306330

6	13.499589760	14.976577590	7.810003330
6	12.277271760	17.117670590	7.843762330
6	11.053555760	19.258797590	7.876510330
6	9.827325760	21.400675590	7.907304330
6	8.596852760	23.551425590	7.937760330
6	19.630533760	8.552557590	7.789706330
6	18.410950760	10.696216590	7.805154330
6	17.188559760	12.837959590	7.817223330
6	15.966513760	14.979142590	7.832234330
6	14.744470760	17.120078590	7.852527330
6	13.520597760	19.261217590	7.881230330
6	12.293641760	21.403834590	7.912305330
6	11.062076760	23.556813590	7.940514330
6	22.093178760	8.550071590	7.824191330
6	20.873419760	10.695589590	7.855083330
6	19.652908760	12.838758590	7.869565330
6	18.429810760	14.979682590	7.882707330
6	17.207970760	17.121914590	7.889924330
6	15.984444760	19.264177590	7.897624330
6	14.757719760	21.408006590	7.920294330
6	13.526775760	23.562243590	7.947610330
6	24.554933760	8.548927590	7.841671330
6	23.333666760	10.694595590	7.881357330
6	22.113631760	12.838896590	7.891342330
6	20.890976760	14.979528590	7.889807330
6	19.670617760	17.122266590	7.930211330
6	18.446284760	19.265174590	7.942596330
6	17.221142760	21.411466590	7.940096330
6	15.991474760	23.567096590	7.956635330
6	27.017122760	8.547975590	7.838714330

6	25.793507760	10.693401590	7.880180330
6	24.571888760	12.837242590	7.870899330
6	23.350015760	14.976121590	7.764933330
6	22.130765760	17.120221590	7.863331330
6	20.908234760	19.263388590	7.972296330
6	19.683990760	21.412234590	7.975285330
6	18.456425760	23.570194590	7.970557330
6	29.479602760	8.547724590	7.827960330
6	28.255002760	10.692201590	7.857173330
6	27.031865760	12.836689590	7.867517330
6	25.814861760	14.976736590	7.784146330
6	24.592777760	17.123470590	7.751322330
6	23.369536760	19.265868590	7.922862330
6	22.146535760	21.410990590	7.986882330
6	20.920664760	23.570881590	7.984628330
6	31.942548760	8.549560590	7.828670330
6	30.716614760	10.692466590	7.833426330
6	29.493449760	12.835621590	7.851544330
6	28.270732760	14.978004590	7.851389330
6	27.049890760	17.120423590	7.832888330
6	25.827816760	19.260668590	7.891957330
6	24.606988760	21.408763590	7.953182330
6	23.383434760	23.569694590	7.976404330
6	34.405922760	8.552924590	7.840148330
6	33.179063760	10.694216590	7.830807330
6	31.955562760	12.836042590	7.830914330
6	30.733726760	14.977730590	7.846828330
6	29.510585760	17.119298590	7.861666330
6	28.288028760	19.262254590	7.879173330
6	27.066808760	21.408143590	7.914825330

6	25.845307760	23.567562590	7.944900330
6	36.870129760	8.555308590	7.853131330
6	35.642534760	10.695990590	7.840734330
6	34.418864760	12.837140590	7.829658330
6	33.196911760	14.978661590	7.827329330
6	31.974322760	17.120175590	7.838118330
6	30.750716760	19.262360590	7.853675330
6	29.528108760	21.407855590	7.875968330
6	28.306225760	23.565051590	7.905668330
6	39.336959760	8.552756590	7.869126330
6	38.107760760	10.694175590	7.851665330
6	36.883684760	12.836720590	7.837937330
6	35.661520760	14.979434590	7.827040330
6	34.438756760	17.121998590	7.823346330
6	33.214236760	19.264427590	7.830477330
6	31.989205760	21.408890590	7.847031330
6	30.765854760	23.564865590	7.871934330
6	41.796242760	8.530767590	7.889740330
6	40.565325760	10.681504590	7.866410330
6	39.341106760	12.825428590	7.847159330
6	38.118181760	14.969186590	7.832767330
6	36.894053760	17.114635590	7.823092330
6	35.667110760	19.261366590	7.821490330
6	34.438944760	21.409822590	7.830099330
6	33.214132760	23.565848590	7.847990330
1	13.764535760	6.564853590	7.697657330
1	12.543173760	8.712450590	7.716996330
1	11.315631760	10.861165590	7.738562330
1	10.088765760	13.008878590	7.766500330
1	8.863188760	15.155544590	7.802586330

1	7.638771760	17.298061590	7.841640330
1	6.413546760	19.438515590	7.876775330
1	5.184224760	21.584928590	7.910208330
1	34.170761760	24.107335590	7.842222330
1	35.392381760	21.958429590	7.826491330
1	36.619961760	19.809952590	7.819605330
1	37.847275760	17.662066590	7.822201330
1	39.072387760	15.514403590	7.832720330
1	40.295513760	13.370359590	7.848313330
1	41.518826760	11.228637590	7.869986330
1	42.746614760	9.081408590	7.895513330
1	32.024451760	25.352599590	7.869400330
1	29.556987760	25.349969590	7.900236330
1	27.090459760	25.352389590	7.934708330
1	24.622360760	25.354955590	7.964120330
1	22.157707760	25.356234590	7.982785330
1	19.690754760	25.355892590	7.986177330
1	17.223797760	25.353410590	7.982954330
1	14.757704760	25.349282590	7.975170330
1	12.292437760	25.344449590	7.966532330
1	9.827311760	25.339516590	7.962876330
1	7.357483760	25.337515590	7.963243330
1	5.197217760	24.083516590	7.946199330
1	15.910648760	5.319750590	7.704057330
1	18.378109760	5.322061590	7.730863330
1	20.844912760	5.319948590	7.754281330
1	23.312367760	5.317439590	7.772013330
1	25.776423760	5.316229590	7.786474330
1	28.241838760	5.315569590	7.799240330
1	30.708905760	5.317131590	7.813918330

1	33.173927760	5.320654590	7.831369330
1	35.638059760	5.324353590	7.851463330
1	38.102732760	5.327868590	7.873680330
1	40.572099760	5.329030590	7.897502330
1	42.732625760	6.582330590	7.910713330
8	25.320798760	15.415470590	12.446740330
8	24.502086760	13.531967590	10.685531330
8	26.139480760	17.298903590	14.207884330
8	27.767209760	14.263981590	12.540952330
58	25.726984760	13.115912590	12.639037330
58	27.360873760	16.563454590	12.348662330
8	23.689534760	18.130929590	10.300879330
8	24.505621760	13.851430590	14.498324330
8	26.135945760	16.979440590	10.395091330
58	24.095750760	15.831441590	10.493241330
8	22.055675760	14.683456590	10.591319330
8	23.693068760	18.450392590	14.113673330
8	20.427976760	17.718448590	12.258316330
8	22.874387760	16.566959590	12.352528330
58	24.099284760	16.150903590	14.306034330
58	20.834161760	15.418890590	12.450613330
58	22.468050760	18.866432590	12.160239330
8	22.059210760	15.002919590	14.404112330
